# The Use of Bone Void Fillers May Not Impact Outcomes After Medial Opening Wedge High Tibial Osteotomy: A Systematic Review

**DOI:** 10.1002/ars2.70017

**Published:** 2026-07-05

**Authors:** Travette Daniel, Stephen Medearis, Emily Jones, Victoria K. Ierulli, Mary K. Mulcahey

**Affiliations:** ^1^ Rosalind Franklin University of Medicine and Science North Chicago Illinois U.S.A.; ^2^ School of Medicine Tulane University New Orleans Louisiana U.S.A.; ^3^ Department of Orthopaedic Surgery and Rehabilitation Loyola University Medical Center Maywood Illinois U.S.A.

## Abstract

**Purpose:**

To examine whether the use of bone void filler significantly influences outcomes of medial opening wedge high tibial osteotomy (OWHTO), with a specific emphasis on gap sizes less than 10 mm.

**Methods:**

A systematic review of the literature was performed according to Preferred Reporting Items for Systematic Reviews and Meta‐Analysis guidelines. Databases searched included PubMed, MedLine, EMBASE, and Web of Science. Studies were included if they met the following criteria: patients treated by OWHTO, clearly described surgical technique (including osteotomy gap size), filler type (synthetic, allograft, autograft), fixation type (locking versus nonlocking plate), type of plate (e.g., Tomofix, Puddu, Orthomed), and the degree of varus correction. The levels of evidence of the included studies ranged from Level I to Level IV according to the Oxford Centre for Evidence‐Based Medicine classification. The studies must have also been reported in English, peer‐reviewed, and originated in the United States or countries that offer the same procedures, protocol, and outcome reporting.

**Results:**

Twenty‐eight of 253 identified studies met inclusion criteria. A total of 1640 knees underwent OWHTO in the 28 studies: 598 (36.4%) had allograft, 488 (29.8%) had no bone void filler, 359 (21.9%) had synthetic, and 195 (11.9%) had autograft. Of the total patients, 963 (58.7%) were women and 676 (41.3%) were men. Different types of bone void fillers were used in each study, with no filler being most common (13/28), followed by allograft (12/28). Complications such as nonunion, loss of correction, infection, and lateral hinge fracture were reported across studies, although the overall rates were low in all groups. Most investigations described satisfactory healing regardless of whether a bone void filler was used. Several studies noted that outcomes in procedures performed without bone void filler, particularly when the osteotomy gap was less than 10 mm, were comparable to or slightly better than those in which a filler was used.

**Conclusions:**

This study revealed that the utilization or absence of bone void filler does not impact the outcomes of OWHTO. In the setting of OWHTO with gap sizes less than 10 mm, placement of a bone void filler may contribute to higher complication rates. Despite the lack of functional advantage of using a bone void filler, OWHTO is still completed with bone void filler in more than 50% of cases.

**Level of Evidence:**

Level IV, systematic review of Level I to IV studies.

## Graphical abstract

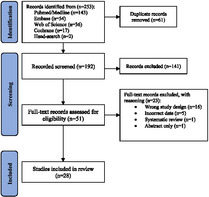

Medial opening wedge high tibial osteotomy (OWHTO) is a well‐established treatment option for the correction of pathologic varus‐aligned knees with osteoarthritis and/or pain.^1^ This procedure has a high success rate with positive patient outcomes, high union rates leading to proper bone healing, predictable correction, and avoidance of injury to adjacent knee structures. However, using an opening wedge technique creates a temporary void within the patient's bone at the osteotomy site, which may lead to delayed union or nonunion if not properly managed.^2^ Several bone void fillers are utilized by orthopaedic surgeons to fill this gap.

Historically, iliac crest autograft was used to fill the bone gap following OWHTO.^3^ An autograft provides a strong structural framework for bony ingrowth with osteogenic properties; however, it can cause increased operative time, donor‐site pain, and bleeding.^4^ An allograft provides the same osteoconductive properties, but does not have the same osteogenic capabilities and results in slow bone formation. Allograft is now the most commonly used bone void filler, but there is a small risk associated with immune rejection and disease transmission.^5^ Many synthetic bone void filler options have been introduced as a supporting material for augmentation after osteotomy without the limited availability and donor site morbidity associated with autograft. These can provide the same functionality as autograft or allograft bone void filler while also serving as a framework for cellular migration and structural support without the previously mentioned complications.^6^

There has been growing evidence that similar healing can be achieved by utilizing locked plate fixation while leaving the gap unfilled. In 2018, Nha et al. reported that in 38 opening wedge high tibial osteotomies with gaps ranging from 7 to 15 mm, bone union was achieved without utilization of a bone graft.^7^ The authors found that OWHTO performed without bone graft showed significantly more incorporation than the synthetic bone graft group within 2 years. Dornacher et al. identified that a gap of greater than 13 mm was a risk factor for nonunion and bone healing disruption in 101 patients managed without bone voidfiller.^8^ The authors used regression analysis to show a positive correlation between the gap size of the opening wedge osteotomy and delayed bone healing. This effect was notably exacerbated when the gap size exceeded 13 mm.^8^ Understanding the optimal technique for achieving gap healing following OWHTO is critical to increasing the likelihood of successful outcomes following this procedure. The purpose of this study was to examine whether the use of bone void filler significantly influences outcomes of medial OWHTO, with a specific emphasis on gap sizes less than 10 mm. We hypothesized that patients administered bone void fillers for OWHTO will experience no significant functional benefit compared with patients who do not receive bone fillers.

## METHODS

### Inclusion and Exclusion Criteria

**FIGURE 1:**
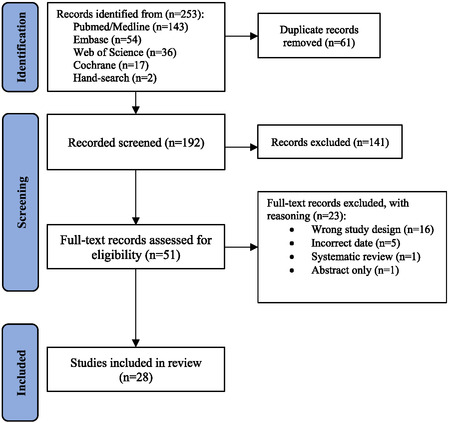
Preferred Reporting Items for Systematic Reviews and Meta‐Analysis (PRISMA) flow chart of the article selection.

A systematic review of the literature was performed according to Preferred Reporting Items for Systematic Reviews and Meta‐Analysis guidelines (Figure 1).^9^ Databases searched included PubMed, Medine, EMBASE, and Web of Science. The following keywords/search terms were included in the search: high tibial osteotomy, medial opening wedge high tibial osteotomy, bone filler, and bone replacement material. Full search syntax is provided in Table 1. The participant, interventions, comparators, and outcomes framework was utilized to determine appropriate studies for inclusion. Studies were included if they met the following criteria: patients treated by OWHTO clearly described surgical technique (including osteotomy gap size), filler type (synthetic, allograft, autograft), fixation type (locking versus nonlocking plate), type of plate (e.g., Tomofix, Puddu, Orthomed), and the degree of varus correction. Studies were excluded if they were not peer‐reviewed journal articles or if they were systematic reviews.

**TABLE 1:** Search Syntax

**#**	**Topic**	**Search**
1	OWHTO	((((high tibial osteotomy[Title/Abstract]) OR (opening tibial osteotomy[Title/Abstract])) OR (medial tibial osteotomy[Title/Abstract])) OR (((opening wedge high tibial osteotomy[Title/Abstract]) OR (OWHTO[Title/Abstract])) OR (medial opening wedge high tibial osteotomy[Title/Abstract])))
2	Bone Fillers	(((((((((((bone substitutes[Title/Abstract]) OR (bone filler[Title/Abstract])) OR (Bone Replacement Material[Title/Abstract])) OR (Bone Replacement Materials[Title/Abstract])) OR (Bone fillers[Title/Abstract])) OR (Bone substitute[Title/Abstract])) OR (bone graft[Title/Abstract])) OR (bone void filler[Title/Abstract])) OR (synthetic bone substitute[Title/Abstract])) OR (tibial osteotomy autograft[Title/Abstract])) OR (tibial osteotomy allograft[Title/Abstract]))
3	FULL	#1 AND #2

OWHTO, opening wedge high tibial osteotomy.

### Data Extraction Protocol and Assessment of Study Quality

Two authors (S.M., E.J.) independently extracted data in duplicate to Excel using a prespecified data extraction template based on the Cochrane data extraction sheet. Information collected from each study was 1) the type of OWHTO gap filler used (synthetic, allograft, autograft, no filler), 2) the percentage of OWHTO procedures performed with each type of bone void filler, 3) osteotomy gap size in millimeters or described with hip‐knee‐ankle angle, 4) type of plate, 5) rate of complications after procedure (e.g., nonunion, infection, lateral hinge fracture, loss of correction), 6) follow‐up time (years), and 7) demographic data (sex, body mass index, age). A data quality criteria assessment was performed based on study design. Validity and variability in eligible articles are both characteristics necessary for this review. Authors agreed on the specific study aim as well as inclusion criteria prior to performing analysis to ensure this was not lost. A variety of quality assessment tools were used to determine the susceptibility of bias. For randomized trials and cross‐sectional studies, Cochrane's Quality Assessment Tool and the Joanna Briggs Institute quality tool were used, respectively.

### Statistical Analysis

Given the substantial heterogeneity in study design, patient populations, surgical techniques, and outcome definitions, as well as inconsistent reporting of variance measures (standard deviations, standard errors, or confidence intervals), no formal pooling of data or meta‐analysis was performed. Results from individual studies were summarized descriptively. Continuous variables (age, body mass index, osteotomy gap size, and follow‐up duration) were reported as ranges, with means included when available from the original articles. Categorical variables, including use of bone void filler type (allograft, autograft, synthetic, or no filler) and the occurrence of complications (nonunion, loss of correction, infection, and lateral hinge fracture), were summarized as counts and proportions.

Outcomes were described qualitatively across studies and stratified by bone void filler category and, when reported, by gap size less than 10 mm versus 10 mm or greater. Simple proportions of complications within each bone void filler group and gap size subgroup were calculated to facilitate descriptive comparison only, without formal hypothesis testing or heterogeneity statistics. All data extraction and descriptive calculations were performed using Microsoft Excel (Microsoft, Redmond, WA).

## RESULTS

A total of 1640 patients (963, 58.7% female; 677, 41.3% male) underwent medial OWHTO in the 28 included studies (Tables 1 and 2). Regarding the use of bone void filler, 598 (36.4%) had allograft, 488 (29.8%) had no bone void filler, 359 (21.9%) had synthetic, and 195 (11.9%) had autograft. Across the included studies, rates of nonunion and infection varied only slightly between groups and did not appear to differ meaningfully based on the use of bone void filler. Reported nonunion rates generally ranged from 0% to 26.3%, and infection rates from 2% to 11.4% (Tables 2 and 3). Several studies noted a higher frequency of lateral hinge fractures in procedures performed without bone void filler, with reported ranges from approximately 0% to 29.3%, although these findings were not consistently associated with worse clinical outcomes. Tables 4 and 5 report the study characteristics.

**TABLE 2:** Summary of Complications Following OWHTO Randomized Control Trial (RCT)

**Author**	**Type of Graft**	**Number of Patients**	**No Complication**	**Nonunion**	**Loss of Correction**	**Infection**	**Lateral Hinge Fracture**	**Level of Evidence**
D Lee et al.^10^	Allograft	27						Level I
Haghpanah et al.^11^	Allograft	23		‐	‐	4.3%	‐	Level II
Chen et al.^12^	Allograft	88	No complication					Level II
Haghpanah et al.^11^	Autogenous	23		‐	‐	‐	‐	Level II
Fucentese et al.^13^	Autogenous	15		‐	‐	‐	20.0%	Level II
Ekeland et al.^14^	Autogenous	49		2.0%	‐	4.1%	‐	Level II
Ulucaokoy et al.^15^	Autogenous	25	No complication					Level II
Bodenbeck et al.^16^	Autogenous	14	No complication					Level II
Darees et al.^17^	No filler	51		3.9%	2.0%	3.9%		Level II
Ferner et al.^18^	No filler	30		3.3%	53.3%	‐	‐	Level II
Kim SJ et al.^6^	No filler	49		‐	‐	‐	6	Level II
Lansdaal et al.^19^	No filler	50		4.0%	0	2.0%	0	Level I
Bodenbeck et al.^16^	No filler	21						Level II
Fucentese et al.^13^	No filler	25		‐	‐	‐	24.0%	Level II
Choi et al.^20^	Synthetic	25	No complication					Level II
Ferner et al.^18^	Synthetic	19		26.3%	68.4%	‐	‐	Level II
D Lee et al.^10^	Synthetic	27	No complication					Level I
Kawashima et al.^21^	Synthetic	39	No complication					Level II
Kobayashi et al.^22^	Synthetic	61		‐	‐	‐	23.0%	Level II
Putnis et al.^23^	Synthetic	15	No complication					Level II

OWHTO, opening wedge high tibial osteotomy.

**TABLE 3:** Summary of Complications Following OWHTO Non‐Randomized Control Trial (NRCT)

**Author**	**Type of Graft**	**Number of Patients**	**No Complication**	**Nonunion**	**Loss of Correction**	**Infection**	**Lateral Hinge Fracture**	**Level of Evidence**
Kim SC et al.^24^	Allograft	39						Level III
O Lee et al.^25^	Allograft	41						Level III
Jeon et al.^26^	Allograft	27						Level III
Kim HJ et al.^27^	Allograft	17						Level III
Koh et al.^28^	Allograft	114		0.9%		11.4%	5.3%	Level III
Villate et al.^29^	Allograft	64			4.7%		4.7%	Level IV
Yazdi et al.^30^	Allograft	102			2.0%	2.0%		Level V
Zhong et al.^31^	Allograft	30	No complication					Level III
Mao et al.^32^	Allograft	24			20.8%	4.2%		Level III
Jung et al.^33^	Autogenous	40		‐	‐	‐	25.0%	Level III
Kim HJ et al.^27^	Autogenous	19	No complication					Level III
Mao et al.^32^	Autogenous	22			4.5%			Level III
Nha et al.^7^	No filler	38		‐	‐	2.6%	5.3%	Level III
Kim SC et al.^24^	No filler	39	No complication					Level III
Park et al.^34^	No filler	73		‐	‐	‐	23.3%	Level III
Jung et al.^33^	No filler	43		‐	‐	4.7%	14.0%	Level III
Siboni et al.^35^	No filler	41		12.2%	‐	‐	29.3%	Level III
Mao et al.^32^	No filler	22			9.1%			Level III
Zhong et al.^31^	No filler	35						Level III
Nha et al.^7^	Synthetic	33		‐	‐	6.1%		Level III
Kim SC et al.^24^	Synthetic	29						Level III
O Lee et al.^25^	Synthetic	41	No complication					Level III
Jung et al.^33^	Synthetic	43		‐	‐	2.3%	18.6%	Level III
Jeon et al.^26^	Synthetic	27	No complication					Level III

OWHTO, opening wedge high tibial osteotomy.

**TABLE 4:** Characteristics of Included Studies Randomized Control Trial (RCT)

**Author**	**Study Design**	**Level of Evidence**	** Number of Patients**	**Average Age**	**Complications**	**Follow‐Up Length**
D Lee et al.^10^	RCT	Level I	54	52	None reported (allograft, synthetic)	12 mo
Haghpanah et al.^11^	RCT	Level II	46	26	One infection (allograft); none (autogenous)	8 yr
Chen et al.^12^	RCT	Level II	82	61	No complications (allograft)	12 mo
Fucentese et al.^13^	RCT	Level II	40	42	Three lateral hinge fractures (autogenous); 6 lateral hinge fractures (no filler)	12 mo
Ekeland et al.^14^	RCT	Level II	49	47	One no complication, 2 loss of correction (autogenous)	8.3 yr
Ulucaokoy et al.^15^	RCT	Level II	25	41	No complications (autogenous)	2.5 yr
Bodenbeck et al.^16^	RCT	Level II	35	44	No complications (autogenous, no filler)	12 mo
Darees et al.^17^	RCT	Level II	48	56	Two nonunion, 1 loss of correction, 2 infections (no filler)	10 yr
Ferner et al.^18^	RCT	Level II	49	44	One nonunion, 16 loss of correction (no filler); 5 nonunion, 13 loss of correction (synthetic)	8 mo
Kim SJ et al.^6^	RCT	Level II	49	52	Six lateral hinge fractures (no filler)	2 yr
Lansdaal et al.^19^	RCT	Level I	50	54	Two nonunion, 1 infection, 0 lateral hinge fracture (no filler)	12 mo
Choi et al.^20^	RCT	Level II	28	58	No complications (synthetic)	12 mo
Kawashima et al.^21^	RCT	Level II	39	58	No complications (synthetic)	4 yr
Kobayashi et al.^22^	RCT	Level II	66	65	Fourteen lateral hinge fractures (synthetic)	6 yr
Putnis et al.^23^	RCT	Level II	15	50	No complications (synthetic)	4 yr

mo, month; RCT, randomized control trial; yr, years.

**TABLE 5:** Characteristics of Included Studies Non‐Randomized Control Trial (NRCT)

**Author**	**Type of Graft**	**Graft Material**	**Patient Total**	**Male**	**Female**	**Average Age**	**Average BMI**	**Gap Size, mm**	**Pre‐OLA (HKA Angle)**	**Post‐OLA (HKA Angle)**	**Pre‐OLA (FTA Varus)**	**Post‐OLA (FTA Valgus)**	**Plate Type**	**Follow‐Up**	**Date of Study**
Kim SC et al.^24^	Allograft	Bone chips	29	11	18	57.79	26.45	11.7							
O Lee et al.^25^	Allograft	Bone chips	53	15	38	52	26.4	10.7							
Jeon et al.^26^	Allograft	Bone chips	27	6	21	56.6	26.5	15							
Kim HJ et al.^27^	Allograft	Bone chips	17	4	13	58.9	24.6	14.4							
Koh et al.^28^	Allograft	Femoral head	114	56	58	54.5 +/− 8	29.25						HTO	Varies	2013‐2020
Villate et al.^29^	Allograft	Osteopure hip	64	43	21	51.8	27.2 +/− 6.4		173	180.9			Surfix	7.5 yr	2004‐2015
Yazdi et al.^30^	Allograft	Iliac crest	102	44	58	40.85	26 +/− 7	9.8					Tomofix	33 mo	2013‐2020
Zhong et al.^31^	Allograft	Bone chips	30	17	13	53.27	25.99	7.39	−5.08	1.35	179.7	172.88	Tomofix	1 yr	2018‐2020
Mao et al.^32^	Allograft	Tibia plateau	24	11	13	50.4	26.8	12.7					Aplus	1 yr	2019‐2021
Jung et al.^33^	Autogenous	Medial femoral condyle	40	8	32	60.7	24	8.1							
Kim HJ et al.^27^	Autogenous	Anterior iliac spine	19	3	16	57.2	24.9	14					Tomofix	1 yr	2011‐2014
Mao et al.^32^	Autogenous	Iliac crest	22	11	11	54.2	26.2	11.4							
Nha et al.^7^	No filler		38	5	33	58.3	26.3	10.5	−6.0 +/− 2.6	2.6 +/− 2.5			Tomofix	2 yr	2012‐2015
Kim SC et al.^24^	No filler		39	6	33	57.15	26.33	11.1					Lcfit HTO	1 yr	2017‐2018
Park et al.^34^	No filler		73	24	49	56.5	27	9.6					Tomofix synthes	13.5 mo	2015‐2018
Jung et al.^33^	No filler		43	9	34	61.5	25.2	8.3					Tomofix	1 yr	2014‐2017
Siboni et al.^35^	No filler		41	20	21	59	30.3		173	183			Tomofix	6 mo	2010‐2015
Zhong et al.^31^	No filler		35	22	13	50.86	26.03	7.19	−4.74	1.5	179.09	172.87			
Mao et al.^32^	No filler		22	10	12	51.2	26.1	11.9							
Nha et al.^7^	Synthetic	HA + β‐TCP	33	11	22	55.9	27.7	11	−6.0 +/− 2.6	2.6 +/− 2.5					
Kim SC et al.^24^	Synthetic		29	10	19	53.03	27.36	11.79							
O Lee et al.^25^	Synthetic	β‐TCP	41	12	29	48.7	26.7	11.1					N/A	1 yr	2014‐2015
Jung et al.^33^	Synthetic	β‐TCP	43	9	34	59.6	24.1	9.1							
Jeon et al.^26^	Synthetic	β‐TCP	27	6	21	54.4	27.8	14.7					Tomofix	1 yr	2014‐2018

BMI, body mass index; FTA, femorotibial angle; HA, hydroxyapatite; HKA, hip‐knee‐ankle angle; HTO, high tibial osteotomy; LCfit, lateral cortex fit; mo, month; N/A, not applicable; OLA, optimal limb alignment; TCP, tricalcium phosphate; yr, year.

Twenty of the 28 studies (71.4%) used the Tomofix plate, whereas 2 (7.1%) used the Orthofix and Surfix plates. The remaining studies used a study specific plate. The age range of the patient population across all 24 studies was 26 to 72 years old.

Thirteen of the 28 studies (46.4%) used no bone void filler,^6^, ^7^, ^13^, ^16^, ^17^, ^18^, ^19^, ^24^, ^31^, ^32^, ^33^, ^34^, ^35^ 12 (42.8%) used allograft,^10^, ^11^, ^12^, ^24^, ^25^, ^26^, ^27^, ^28^, ^29^, ^30^, ^31^, ^32^ 11 (39.3%) used synthetic bone void filler,^7^, ^10^, ^18^, ^20^, ^21^, ^22^, ^23^, ^24^, ^25^, ^26^, ^33^ and 8 (28.6%) used autograft (Table 3).^3^, ^11^, ^13^, ^14^, ^15^, ^16^, ^27^, ^32^ Thirteen of the 28 studies (46.4%) compared outcomes of multiple bone void fillers simultaneously, including a variety of age groups, body mass indices, and several different follow up times (Table 2).

The complications observed across all the studies included nonunion in 17 patients, loss of correction in 35 patients, infection in 28 patients, and lateral hinge fracture in 93 patients. The most common complication was lateral hinge fracture, which occurred in 93 patients. Several studies noted that lateral hinge fractures were more frequently reported in the group without bone void filler (Table 4 and 5). Patients in this group also tended to fall within higher age and body mass index ranges compared with those who received bone void fillers.

Several individual studies indicated unexpected findings regarding the choice of bone void filler. In 2015, Park et al. evaluated 73 patients who underwent OWHTO with an average gap size of 9.6 +/− 2.8 mm who did not receive bone void filler.^34^ The authors found no complications between the groups that had a correction angle greater than and less than 10° after 13.5 months. Kim et al. evaluated 97 total patients, 39 who underwent OWHTO with no bone void filler, 29 with synthetic bone void filler, and 29 with allograft.^24^ The authors found no complications among any of the 97 patients after 1 year. Siboni et al. evaluated 41 patients who underwent OWHTO without bone void filler. The authors found that 5 of the 41 patients developed a nonunion over 6 months, which accounted for 50% of the nonunion cases among the 488 patients in the no‐filler group.^35^

The methodological quality of the included studies was low and heterogenous resulting in low statistical power. Fourteen out of 28 (50.0%) studies were case series, without direct comparison between bone void filler types, making it difficult to reliably compare outcomes with statistical significance.

We conducted a risk of bias assessment using Methodolgical Index for Non‐Randomized Studies criteria and determined that our study carries a moderate risk of bias, as shown in Table 6.

**TABLE 6:** MINORS Criteria

**MINORS Criteria**	**Lobenhoffer P** ^1^	**Yoon JR** ^2^	**Han JH, Kim HJ, Song JG, et al.** ^3^	**Chae DJ, Shetty GM, Wang KH, Montalban AS, Jr., Kim JI, Nha KW** ^4^	**Bauer TW, Muschler GF** ^5^	**Kim SJ, Nguyen LT, Seo YJ, et al.** ^6^	**Nha KW, Oh SM, Ha YW, et al.** ^7^	**Dornacher D, Leitz F, Kappe T, Reichel H, Faschingbauer M** ^8^	**Page MJ, McKenzie JE, Bossuyt PM, et al.** ^9^	**Darees M, Putman S, Brosset T, Roumazeille T, Pasquier G, Migaud H** ^17^
A clearly stated aim	2	2	2	2	2	2	2	2	2	2
Inclusion of consecutive patients	1	0	1	1	0	2	1	2	0	2
Prospective data collection	1	0	0	1	0	2	0	1	0	1
Endpoints appropriate to the aim of the study	2	1	2	2	2	2	2	2	2	2
Unbiased assessment of the study endpoints	1	0	2	1	1	1	1	1	1	1
Follow‐up period appropriate to the aim of the study	2	0	1	1	0	2	2	2	0	2
Loss to follow‐up less than 5%	1	0	1	1	0	1	1	1	0	1
Prospective calculation of the study size	0	0	0	0	0	0	0	0	0	0
Adequate control group	0	0	2	0	0	0	2	0	0	0
Contemporary groups	0	0	1	0	0	0	1	0	0	0
Baseline equivalence of groups	1	0	2	2	0	2	2	2	0	2
Adequate statistical analysis	2	1	2	2	2	2	2	2	2	2
Results	13/16	4/16	16/24	13/16	7/16	16/16	16/24	15/16	7/16	15/24

**MINORS Criteria**	**Ferner F, Dickschas J, Ostertag H, et al.** ^18^	**Park HJ, Kang SB, Chang MJ, Chang CB, Jung WH, Jin H** ^34^	**Jung WH, Takeuchi R, Kim DH, Nag R** ^33^	**Fucentese SF, Tscholl PM, Sutter R, Brucker PU, Meyer DC, Koch PP** ^13^	**Lansdaal JR, Mouton T, Wascher DC, et al.** ^19^	**Zhong R, Yu G, Wang Y, et al.** ^31^	**Mao Y, Yao L, Li J, Li J, Xiong Y** ^32^	**Bodenbeck E‐M, Böpple JC, Doll J, Bürkle F, Schmidmaier G, Fischer C** ^16^		**Lee DY, Lee MC, Ha CW, et al.** ^10^
A clearly stated aim	2	2	2	2	2	2	2	2	2	2
Inclusion of consecutive patients	1	2	2	2	2	1	1	1	1	2
Prospective data collection	1	1	1	2	2	0	0	0	0	2
Endpoints appropriate to the aim of the study	2	2	2	2	2	2	2	2	2	2
Unbiased assessment of the study endpoints	1	1	1	2	2	1	1	1	1	2
Follow‐up period appropriate to the aim of the study	2	2	2	2	2	2	2	2	2	2
Loss to follow‐up less than 5%	1	1	1	1	1	0	0	0	0	2
Prospective calculation of the study size	0	0	0	2	2	0	0	0	0	2
Adequate control group	2	0	2	2	2	2	2	2	0	2
Contemporary groups	1	0	1	2	2	1	1	1	0	2
Baseline equivalence of groups	2	2	2	2	2	1	1	1	1	2
Adequate statistical analysis	2	2	2	2	2	2	2	2	2	2
Results	17/24	15/16	18/24	23/24	23/24	14/24	14/24	14/16	11/16	24/24

**MINORS Criteria**	**Kim HJ, Seo I, Shin JY, Lee K.S., Park KH, Kyung HS** ^27^	**Haghpanah B, Kaseb MH, Espandar R, Mortazavi SMJ** ^11^	**Lee OS, Lee KJ, Lee YS** ^25^	**Jeon JW, Jang S, Ro DH, Lee MC, Han HS** ^26^	**Koh DTS, Lee KH** ^28^	**Villatte G, Erivan R, Fournier PL, et al.** ^29^	**Yazdi HR, Karimi Haris H, Rohani S, Karimi N** ^30^	**Chen J, Li J, Zhang H, et al.** ^12^	**Choi WC, Kim B., Kim U, Lee Y, Kim JH** ^20^	**Kawashima F, Takagi H** ^21^
A clearly stated aim	2	2	2	2	2	2	2	2	2	2
Inclusion of consecutive patients	1	2	1	1	1	1	1	1	1	1
Prospective data collection	0	2	0	0	0	0	0	1	1	1
Endpoints appropriate to the aim of the study	2	2	2	2	2	2	2	2	2	2
Unbiased assessment of the study endpoints	1	2	1	1	1	1	1	1	1	1
Follow‐up period appropriate to the aim of the study	2	2	2	2	2	2	2	2	2	2
Loss to follow‐up less than 5%	0	2	0	0	0	1	1	1	1	1
Prospective calculation of the study size	0	2	0	0	0	0	0	0	0	0
Adequate control group	2	2	2	2	2	1	1	1	1	1
Contemporary groups	1	2	1	2	2	0	0	0	0	0
Baseline equivalence of groups	2	2	2	2	2	2	2	2	2	2
Adequate statistical analysis	2	2	2	2	2	2	2	2	2	2
Results	15/24	24/24	15/16	16/16	16/16	14/16	14/16	15/16	15/16	15/16

**MINORS Criteria**	**Kobayashi H, Akamatsu Y, Kumagai K, Kusayama Y, Saito T** ^22^	**Putnis S, Neri T, Klasan A, Coolican M** ^23^	**Ekeland A, Nerhus TK, Dimmen S, Thornes E, Heir S** ^14^	**Ulucaköy C, Yapar A, Vural A, Özer H** ^15^	**Slevin O, Ayeni OR, Hinterwimmer S, Tischer T, Feucht M.J, Hirschmann MT** ^36^	**Mao Y, You M, Zhang L, Li J, Fu W** ^37^	**Goshima K, Sawaguchi T, Shigemoto K, et al.** ^38^	**Lee SS, Celik H, Lee DH** ^39^
A clearly stated aim	2	2	2	2	2	2	2	2
Inclusion of consecutive patients	1	1	1	1	0	0	2	2
Prospective data collection	1	1	2	1	0	0	1	1
Endpoints appropriate to the aim of the study	2	2	2	2	2	2	2	2
Unbiased assessment of the study endpoints	1	1	1	1	2	2	2	2
Follow‐up period appropriate to the aim of the study	2	2	2	2	2	2	2	1
Loss to follow‐up less than 5%	1	1	1	1	0	0	1	1
Prospective calculation of the study size	0	0	0	0	0	0	0	0
Adequate control group	1	1	0	2	2	2	0	2
Contemporary groups	0	0	0	2	0	0	0	0
Baseline equivalence of groups	2	2	2	2	2	2	2	2
Adequate statistical analysis	2	2	2	2	2	2	2	2
**Results**	12/16	15/16	15/16	18/24	14/16	14/16	16/16	17/24

MINORS, Methodological Index for Non‐Randomized Studies.

## DISCUSSION

The findings of this study suggest there is no considerable difference in subjective outcomes following medial OWHTO with or without the use of bone void filler. Similar complication rates occurred across all groups and there was no difference in complication rates of bone void filler compared with no filler. One case of nonunion occurred among 195 patients who underwent OWHTO without bone void filler and a gap size less than 10 mm. In comparison, 6 cases of nonunion occurred among 347 patients who underwent OWHTO with bone void filler and had a gap size less than 10 mm. However, among OWHTO patients with gap sizes greater than 10 mm, the rate of nonunion was highest among the nonfiller group. Both of these comparisons lacked statistical significance; however, the infrequent occurrence of complications across all treatment choices suggests that the outcomes are independent of whether bone void filler is used.

Our studies varied in follow‐up time from 6 months to 10 years and increased complication rates were seen with longer follow‐up. For this reason, the rate of complications may not be a reliable measure of difference in outcomes across different types of bone void fillers. To mitigate these limitations, we conducted our search using multiple databases and reported our results based on the comprehensive patient outcomes observed across the studies. Even with the heterogeneity in technique showing different complications and outcomes, this data proved useful in cautioning surgeons about possible risks involved across filler type in relation to the patient demographic they operate on.

A 2016 systematic review by Slevin et al. suggested that OWHTO with gap size less than 10 mm could be performed without using a bone void filler at no risk of additional complications.^36^ Prior to this, the literature often suggested that iliac crest autograft was the gold standard.^33^ In this previous systematic review, only 14.6% of patients underwent OWHTO without bone void filler, whereas 45.5% received autograft bone void filler, predominantly from the iliac crest with less sourced from the femoral condyle.^36^ An increase in the overall frequency of OWHTO without bone void filler was also reflected in a systematic review performed by Mao et al. in 2022.^37^ The authors found that there was a lack of improved healing with similar complication rates for bone void fillers when compared with the no‐filler group.

Despite an increase in the number of studies suggesting there is no subjective benefit to filling the gap after OWHTO, there is a body of literature suggesting that using a bone void filler is beneficial anecdotally, particularly when the gap size is greater than 10 mm or in patients considered to have higher risk of nonunion, such as smokers, diabetics, and the elderly. The authors reported that the elasticity of the lateral cortex functions as an essential biomechanical stabilizer during medial opening wedge high tibial osteotomy.^40^ The elastic properties of the lateral cortex permit controlled widening of the osteotomy; however, when the opening gap becomes excessive, typically greater than 10 mm as showed in prior studies, the lateral hinge is subjected to forces beyond its elastic capacity and becomes more susceptible to fractures. The risk is further increased when hinge position, osteotomy technique, or postoperative loading is suboptimal. Consistent with these findings, Lee at al. similarly noted that medial opening wedge osteotomy relies on the lateral cortex's elasticity for stability and that opening the gap beyond this elastic limit significantly elevates the likelihood of lateral hinge fracture. A 2019 study by Goshima et al. followed 93 patients who underwent OWHTO without bone void filler and compared healing via radiographs among patients with different gap sizes.^38^ The authors found that gaps above 13 mm resulted in delayed healing at 6 months when not filled. In 2021, Dornacher et al. retrospectively analyzed 101 of their OWHTO cases performed without a bone void filler and found other risk factors, such as increased opening wedge angle and increased age were associated with delayed healing.^8^ It is possible that the lack of clarity on the necessity of using a bone void filler for gaps >10 mm is due to orthopaedic surgeons’ preference and previous experience. Radiographs provide insight into the degree of healing after OWHTO, but there is substantial variability in interpretation.

It is important to note that lateral hinge fractures were more commonly reported in studies that did not use bone void fillers. However, Dornacher et al. and other studies concluded that lateral hinge fractures have no impact on gap healing.^8^ Lee et al. suggested that as many as 50% of OWHTO may have a lateral hinge fracture when evaluated with a postoperative computed tomography (CT).^39^ Therefore, lateral hinge fracture was not a factor to be considered in the planning of OWHTO, primarily because it did not significantly impact the overall patient outcomes.

Although there is a lack of consensus regarding acceptable osteotomy size without bone void filler, our study suggests OWHTO with gaps larger than 10 mm are at greater risk for lateral hinge fractures. Currently, there is not enough data to determine whether using a bone void filler for large gap OWHTO is necessary to improve healing and decrease the risk of complications.

### Limitations

There are several limitations to this study. The methodological quality of the included studies was low. Thirteen out of 24 (54.1%) studies were case series, without direct comparison between bone void filler types; therefore, it was difficult to reliably compare outcomes with statistical significance. Most of the numerical results were reported by the mean and range without any additional descriptive explanation, which made reporting statistical significance unfeasible. There was substantial variability in the definition of complications associated with OWHTO, particularly with regard to lateral hinge fracture and nonunion. After conducting a risk of bias assessment using Methodological Index for Non‐Randomized Studies criteria, we determined that our study carries a moderate risk of bias, as shown in Table 6. This is primarily due to the majority of the studies being nonrandomized, leading to potential selection bias. Additionally, the follow‐up periods across the studies were varied in length, as well as sample sizes, which may affect the generalizability of the findings. This review was limited by the lack of sex‐disaggregated outcomes in the included studies, which prevented further evaluation in accordance with SAGER guidelines.

## CONCLUSIONS

This study reveals that the utilization or absence of bone void filler does not impact the outcomes of OWHTO. In the setting of OWHTO with gap sizes less than 10 mm, bone void filler may contribute to higher complication rates. Despite the lack of functional advantage of using a bone void filler, OWHTO is still completed with bone void filler in more than 50% of cases. Orthopaedic surgeons should consider this data when using a bone void filler for OWHTO with gap sizes less than 10 mm as its use may not provide meaningful clinical advantages.

## DISCLOSURES

The author (M.K.M.) declares the following financial interests/personal relationships which may be considered as potential competing interests: M.K.M. reports the following: AAOS: Board or committee member; *American Journal of Sports Medicine* Electronic Media: Editorial or governing board; American Orthopaedic Association: Board or committee member American Orthopaedic Society for Sports Medicine: Board or committee member; Arthrex: Paid consultant, paid presenter or speaker; *Arthroscopy*: Editorial or governing board; Arthroscopy Association of North America: Board or committee member; Association of Bone and Joint Surgeons: Board or committee member; International Society of Arthroscopy, Knee Surgery, and Orthopaedic Sports Medicine; Board or committee member; *Journal of Bone and Joint Surgery ‐ American*: Editorial or governing board Ortho Info: Editorial or governing board. The other authors (T.D., S.M., E.J., V.K.I.) declare that they have no known competing financial interests or personal relationships that could have appeared to influence the work reported in this paper.
